# A Retrospective Study of Brain Metastases From Solid Malignancies: The Effect of Immune Checkpoint Inhibitors

**DOI:** 10.3389/fonc.2021.667847

**Published:** 2021-08-27

**Authors:** Wei Du, Cristian Sirbu, B. Daniel Lucas, Steven J. Jubelirer, Ahmed Khalid, Lin Mei

**Affiliations:** ^1^Charleston Area Medical Center (CAMC) Health Education and Research Institute, Charleston, WV, United States; ^2^CAMC Cancer Center, Charleston, WV, United States

**Keywords:** brain metastases, TriNetX database, immune check point inhibitor, immunotherapy, PD-1 inhibitor, PD-L1 inhibitor, CTLA-4 inhibitor

## Abstract

**Introduction:**

Brain metastases (BM) are associated with dismal prognosis, and there is a dearth of effective systemic therapy. In this study, patients with BM from multiple solid tumors were identified from TriNetX databases, their clinicopathological features were evaluated, and the effects of immune checkpoint inhibitor (ICI) therapy were assessed.

**Methods:**

Variables, including median overall survival (OS), Eastern Cooperative Oncology Group (ECOG) performance status, primary diagnosis, and date of diagnosis, were retrieved from TriNetX, a real-world database. Kaplan-Meier plots and log-rank tests were applied to assess significance of differences in survival. Hazard ratio (HR) and 95% confidence interval (CI) values were calculated. All patient data were deidentified.

**Results:**

A total of 227,255 patients with BM were identified in the TriNetX database; median OS was 12.3 months from initial cancer diagnosis and 7.1 months from development of BM. OS of BM from nonsmall-cell lung cancer (NSCLC), triple-negative breast cancer (TNBC), melanoma, and renal cell carcinoma (RCC) were 8.7, 14.7, 17.8, and 15.6 months, respectively. After matching patient baseline characteristics, OS of cohorts with or without exposure to ICIs was evaluated. For all types of cancer, median OS durations for the ICI and no-ICI cohorts were 14.0 and 7.9 months, respectively (HR: 0.88; 95% CI: 0.85–0.91). More specifically, OS was remarkably prolonged in patients with NSCLC (14.4 *vs*. 8.2 months; HR: 0.86; 95% CI: 0.82–0.90), TNBC (23.9 *vs*. 11.6 months; HR: 0.87; 95% CI: 0.82–0.92), and melanoma (27.6 *vs*. 16.8 months; HR: 0.80; 95% CI: 0.73–0.88) if patients had exposure to ICIs. In contrast, there was no significant difference in OS of patients with RCC treated with and without ICIs (16.7 *vs*. 14.0 months; HR: 0.96; 95% CI: 0.86–1.10).

**Conclusions:**

Overall, BM indicates poor patient outcome. Treatment with ICIs improves survival of patients with NSCLC, TNBC, and melanoma and BM; however, no significant improvement was observed in RCC. Investigations to identify prognostic features, oncogenomic profiles, and predictive biomarkers are warranted.

## Introduction

Brain metastases (BM) are estimated to occur in approximately 20% of patients with all types of cancer and are generally associated with poor outcomes (1); however, population-based analysis of prognosis is lacking. A historical cohort study, conducted from 1973 to 2001 in the Detroit metropolitan area, showed that the incidence of all types of cancer was 9.6% (2). According to data from the Surveillance, Epidemiology and End Results (SEER) database, BM was present in 1.7% of cases at diagnosis of cancer from 2010 to 2013 (3). Lung, breast, melanoma, and renal cell carcinoma are the most common types of cancer associated with BM (2, 4). In stage IV nonsmall-cell lung cancer (NSCLC), approximately 10%–25% of cases present with BM at diagnosis and another 10%–30% subsequently develop BM (5, 6). Hence, patients with BM represent a substantial population with unmet needs.

For many years, therapeutic strategies for patients with BM were mainly palliative in nature and failed to improve survival in the majority of cases. For example, in the population with BM when newly diagnosed with cancer after 2010, the median overall survival (OS) durations were only 4.0 and 6.0 months for patients with squamous cell and NSCLC adenocarcinoma, respectively (4), with 6.0 months recorded for those with triple-negative breast cancer (TNBC) (7); there was no significant improvement in outcomes compared with the historical cohort (1973–1993) (8), indicating an urgent need for effective treatments. Although radiotherapy and surgery remain the cornerstones of treatment regimens, emerging new modalities, such as immunotherapy (9, 10) and targeted therapy (11), have slowly improved survival outcomes for patients with several cancer subtypes. In addition, unraveling the biological profiles and driver mutations in BM is crucial to facilitate identification of therapeutic targets. An increasing number of systemic treatment options are becoming available, including human epidermal growth factor receptor 2 (HER2)-targeted therapies and tyrosine kinase inhibitors for NSCLC with driver mutations; however, tumors without druggable mutations lack effective approaches, partially given the molecular divergence of primary tumors and BM, as well as the limitations caused by the blood-brain barrier (6). Immune checkpoint inhibitors (ICIs) have great promise for treatment of all types of cancer, including BM. Therefore, a better understanding of the epidemiology of BM, and particularly comparison of the survival benefit of treatment with or without ICIs, are important to inform tailored therapeutic approaches. Accordingly, the objective of this study was to investigate survival differences of patients with BM treated with and without ICIs and explore the efficacy of immunotherapy using real-world data.

## Materials and Methods

### Ethics Approval

This study was a retrospective analysis of patient data obtained from deidentified databases. The research was conducted in accordance with the Declaration of Helsinki. The protocol was approved by the Institutional Review Board at CAMC (IRB Number: 20-662). For this type of study, formal patient consent was not required.

### Data Source

The TriNetX Research Network (TriNetX Inc., Cambridge, MA) is a real-world and in-house database; it is a global-federated health research network, combining real-time access to longitudinal electronic medical records and administrative claims data. Participating healthcare organizations (HCOs) span patients from a wide range of geographic locations, age groups, and income levels. Details of and use of the network by our team has been described previously (12). The TriNetX platform is Health Insurance Portability and Accountability Act (HIPPA) and General Data Protection Regulation (GDPR) compliant. The majority of contributing HCOs are located in the USA and the European Union.

### Data Collection

Data were retrieved from the Diamond Network subnet, which comprises HCOs contributing online patient information from >200 million individuals. The study period for patients with diagnosis of BM was between January 1st, 2015 and June 30th, 2020, with follow-up until December 31st, 2020 for the primary end point (death). Patients were identified using the ICD-10 code for brain metastasis (C79.3), and primary cancers were also identified using the relevant ICD-10 codes. Only patients ≥18 years old were enrolled. Benign tumors and primary brain tumors were excluded from our study. Baseline demographic characteristics, comorbidities, treatment history, and Eastern Cooperative Oncology Group (ECOG) performance status data were collected. Patients with primary NSCLC, TNBC, melanoma, and RCC were included, which were the tumors most commonly treated with ICIs during the period of the study. Exposure to ICIs was defined as treatment with at least one dose with inhibitors of programmed cell death 1 (PD-1) or its ligand (PD-L1) (nivolumab, pembrolizumab, atezolizumab, avelumab, and durvalumab) or the cytotoxic T-lymphocyte antigen 4 (CTLA-4) inhibitor, ipilimumab. In patients with NSCLC, tumors with driver mutations (of EGFR, ALK, or ROS) were excluded. In the breast cancer cohort, only patients with TNBC for which ICI treatment was indicated were included. In the melanoma cohort, tumors with the BRAF V600E mutation were excluded. For all cohort and patient data, results and patient information were extracted from TriNetX by constructing queries including appropriate ICD-10 codes and procedure codes.

### Data Analysis

Analyses were conducted by the authors. Categorical and continuous parameters were analyzed using Chi-square and analysis of variance (ANOVA), respectively, to determine the statistical significance of differences. Kaplan-Meier plots were generated for univariate analysis comparisons and the log-rank test used to evaluate the significance of differences in OS. For multivariate analysis, Cox proportional hazard regression modeling was employed, based on the results of univariate analyses. Hazard ratio (HR) and 95% confidence interval (CI) values were calculated. To account for differences in baseline characteristics between groups, a propensity score matching (PSM) model was developed using logistic regression to derive well-matched groups for comparative outcomes analysis. Verification was conducted using the nearest-neighbor matching algorithm, with a tolerance level of 0.01 and difference between a propensity score of ≤0.1. GraphPad Prism 6 was used to conduct statistical analysis and generate figures. All tests were two sided, and statistical significance was defined as *p* < 0.05.

## Results

### Overall Survival in Patients With Brain Metastasis

**Figure 1:**
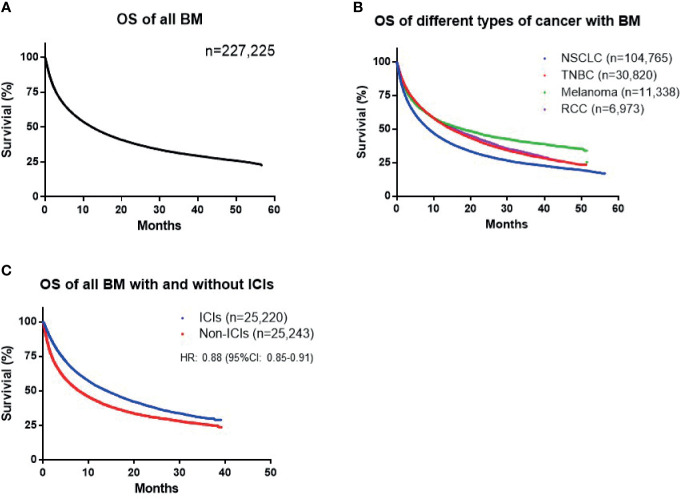
**(A)** Overall survival (OS) of all patients with brain metastases (BM). **(B)** OS of patients with nonsmall-cell lung cancer (NSCLC), triple-negative breast cancer (TNBC), melanoma, and renal cell carcinoma (RCC). **(C)** Difference in survival between cohorts treated with and without immune checkpoint inhibitors (ICIs) among all patients with BM.

A total of 227,255 patients diagnosed with BM between January 1st, 2015 and June 30th, 2020 were identified in the TriNetX database. Of identified cases, 103,248 died before December 31st, 2020, with a median OS of 12.3 months from initial diagnosis of primary cancer and 7.1 months from the development of BM (**Figure 1A**). Furthermore, we analyzed the survival times of patients with different types of cancer. Specifically, patients with NSCLC, TNBC, melanoma, and RCC were investigated, since ICIs were more commonly used to treat these types of tumor. A total of 104,765 patients were diagnosed with NSCLC, and 48,894 reached the primary end point (death). Median OS in patients with NSCLC was significantly shorter than that in patients with malignancies in all sites (8.7 *vs*. 12.3 months; HR: 1.30; 95% CI: 1.28–1.32). A total of 30,820 patients diagnosed with TNBC with BM were identified, with a median OS of 14.7 months. The median OS durations of patients with melanoma (*n* = 11,338) and RCC (*n* = 6,973) were 17.8 and 15.6 months, respectively (**Figure 1B**); all of which represented better than average prognosis.

### Influence of Immune Checkpoint Inhibitors on Overall Survival of Patients With Brain Metastasis

**Figure 2:**
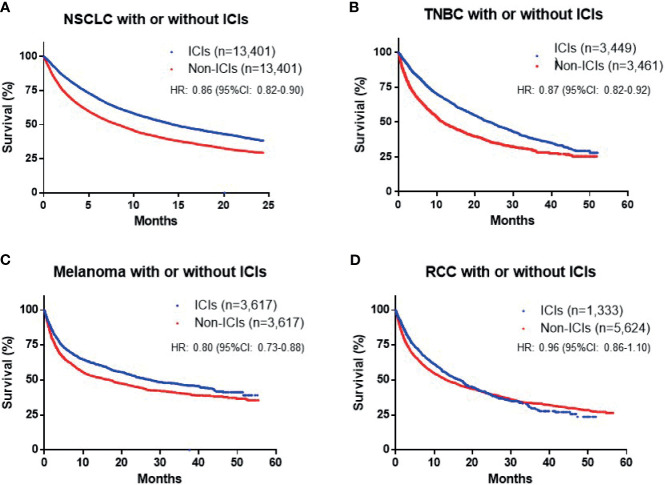
Overall survival (OS) in patients with different cancer subtypes [**(A)** nonsmall-cell lung cancer (NSCLC), **(B)** triple-negative breast cancer (TNBC), **(C)** melanoma and **(D)** renal cell carcinoma (RCC)], with and without immune checkpoint inhibitor (ICI) treatment.

To further investigate the influence of ICIs on patient outcome, we matched patients according to baseline demographic characteristics, comorbidities, prior radiotherapy, and surgery, as well as ECOG performance status using a PSM model (**Table 1**). For all types of cancer, the cohort with ICI exposure included 25,220 patients and the non-ICI-exposed group included 25,243 patients. A total of 37,169 events reached the primary end point. The OS durations of patients in the ICI and no-ICI cohorts were 14.0 *vs*. 7.9 months (HR: 0.88; 95% CI: 0.85–0.91), indicating a significant improvement in survival of patients exposed to ICIs (**Figure 1C**). In the NSCLC group, 13,401 cases were included in each of the ICI and no-ICI cohorts and median OS durations were 14.4 *vs*. 8.2 months, respectively (HR: 0.86; 95% CI: 0.82–0.90). In the TNBC group, 3,449 and 3,461 cases were included in the ICI and no-ICI cohorts, with respective median OS of 23.9 *vs*. 11.6 months (HR: 0.87; 95% CI: 0.82–0.92). Similarly, 3,617 cases with melanoma and BM were included in the ICI and no-ICI cohorts, with median OS of 27.6 *vs*. 16.8 months, respectively (HR: 0.80; 95% CI: 0.73–0.88). These data reveal significant benefits of ICI exposure in these cancer types; however, analysis of patients with RCC with BM, including 1,333 and 5,624 cases in the ICI and no-ICI cohorts, respectively, failed to demonstrate a significant benefit of ICI treatment, with median OS duration of 16.7 *vs*. 14.0 months (HR: 0.96; 95% CI: 0.86–1.10) (**Figures 2A–D**).

**Table 1:** Demographic and clinical characteristics of patients with brain metastases.

Demographic	NSCLC (nondriven mutation)			Melanoma (non-BRAF mutated)			RCC			TNBC		
	ICIs	Non-ICIs	*p*-Value	ICIs	Non-ICIs	*p*-Value	ICIs	Non-ICIs	*p*-Value	ICIs	Non-ICIs	*p*-Value
Number	13,401	13,429		3,617	3,702		1,333	5,624		3,449	3,461	
Age	65.0 ± 10.1	64.8 ± 11	0.18	63.5 ± 13.7	62.6 ± 14.3	0.07	63.7 ± 10.3	63.3 ± 10.8	0.26	59.0 ± 12.4	61.8 ± 12.7	0.33
Sex												
Female	51.6%	52.6%	0.10	32.8%	33.3%	0.74	27.1%	28.5%	0.41	99.8%	99.8%	0.97
Male	48.3%	47.4%	0.10	67.1%	66.6%	0.74	72.8%	71.4%	0.43	0.2%	0.2%	0.83
Race												
White	75.1%	76.0%	0.67	78.7%	78.2%	0.68	71.0%	75.0%	0.36	77.3%	77.1%	0.88
Non-White	24.9%	24.0%	0.65	21.3%	21.7%	0.69	29.0%	25.0%	0.40	22.7%	22.9%	0.61
Smoking	93.2%	94.1%	0.53	46.9%	51.2%	0.08	31.8%	40.3%	0.19	13.2%	12.9%	0.30
Cardiovascular	52.3%	55.6%	0.73	56.2%	55.5%	0.62	67.8%	66.4%	0.23	51.5%	55.4%	0.25
COPD	41.4%	42.6%	0.92	19.4%	19.2%	0.90	10.7%	8.9%	0.08	14.3%	13.6%	0.41
Liver disease	9.9%	9.2%	0.33	21.4%	21.5%	0.97	18.8%	19.0%	0.88	13.4%	12.0%	0.13
ECOG ≥2	15.0%	16.2%	0.17	26.7%	28.9%	0.50	13.1%	11.3%	0.11	32.1%	26.5%	0.21
Brain radiation	62.5%	64.1%	0.26	40.7%	40.5%	0.85	28.1%	27.9%	0.89	37.2%	43.5%	0.19
Brain surgery	10.1%	9.7%	0.21	9.3%	8.7%	0.56	6.2%	5.3%	0.27	6.3%	3.5%	0.46
Chemo/targeted therapy	38.0%	35.2%	0.19	3.5%	2.6%	0.09	33.0%	30.8%	0.33	80.6%	81.4%	0.53

## Discussion

Development of BM usually indicates poor prognosis, with 2- and 5-year OS rates of only 8.1% and 2.4%, respectively, across all types of cancer (13). Patients who present with BM at initial diagnosis have even worse outcomes (3). In this study, we found that median OS of patients with BM was 12.3 months from initial cancer diagnosis and 7.1 months from the development of BM. Compared with a single-center report from the University of Minnesota of a study conducted between 1973 and 1993, which revealed a median OS of approximately 4 months from the development of BM (14), our data indicate very limited improvement in patient outcomes, even with the tremendous changes in antitumor therapies over the intervening period.

Lung cancer, including NSCLC and SCLC, remains the most common type of cancer presenting with BM and accounts for >60% of BM cases (5). Similarly, in our study, NSCLC accounted for 46.1% of total BM cases in the TriNetX database. In addition, 10%–25% of patients with NSCLC may present with BM at initial diagnosis (6) and up to 50% of patients with NSCLC develop BM during the course of their illness (15). This number may continue to rise, due to early screening for BM using brain magnetic resonance imaging. We found that the median OS of patients with NSCLC with BM was only 8.7 months, which was similar to a previous report of approximately 7.0 months, based on analysis of multi-institutional retrospective database between 2006 and 2014 (16). Breast cancer is the second most common type of cancer from which BM develops. A recent report from Martin et al. demonstrated that the median OS of patients with TNBC was approximately 6 months from diagnosis of BM (7) with an OS of 12.5 months for patients with hormone receptor-positive breast cancer (17). With the development of anti-HER2 treatment, patients with HER2-positive breast cancer also achieve significantly superior outcomes compared with their counterparts with TNBC (12 *vs*. 5 months) (6, 7). Melanoma and RCC are also common types of cancer which can develop BM and for which there were no major therapeutic advances in the preimmunotherapy era (18, 19).

A revolution in anti-cancer treatment has occurred since the approval of ipilimumab in 2011. Notably, nivolumab and pembrolizumab have been available since late 2014 and were widely accessible from 2015. Since then, the development of ICIs represents a paradigm shift in oncology therapy and prompted us to further study the role of ICIs in treatment of patients with BM. The Checkmate 204 trial of dual ICI therapy showed a dramatic intracranial response rate of 57% of BM from melanoma (10), as did the randomized phase II ABC trial (20). Hence, dual ICI therapy is established as a cornerstone regimen for patients with small asymptomatic BM from melanoma. In contrast, there is limited evidence supporting the efficacy of PD-1/PD-L1 inhibitor treatment for NSCLC. Goldberg et al. reported an approximately 30% intracranial response rate of treatment with pembrolizumab; however, only in the PD-L1-positive patient cohort (9). In contrast, a population study from Italy reported an intracranial response rate of only 17% (21). Unfortunately, untreated BM from RCC failed to show any response to nivolumab in a phase II trial (22). In addition, there is a lack of evidence for the effectiveness of ICIs for treating BM from TNBC, since approval for their use in this context was only obtained relatively recently (23). The efficacy or benefit of ICIs for BM remains controversial to date. Hence, in our study, we sought to validate previously reported results using real-world data.

Using the TriNetX database, we identified patient cohorts with NSCLC, TNBC, melanoma, and RCC, four types of cancer commonly treated with ICIs during 2015–2020. The cohorts were matched for baseline characteristics, including age, sex, race, cardiovascular disease, lung and liver disease, ECOG performance status, prior brain radiotherapy, brain surgery, chemotherapy, and target therapy, which are important prognostic factors. First, our analysis revealed that exposure to ICIs led to improvement of OS by approximately 6 months for all patients with BM. In subtype studies, we specifically excluded NSCLC with driver mutations and melanoma with BRAF V600E mutation, to avoid bias. The results showed that exposure to ICIs significantly prolonged survival of patients with NSCLC, TNBC, and melanoma (HR: 0.80–0.87); however, no significant therapeutic effect was observed for patients with RCC (HR: 0.96). Amin et al. reported an association between immunotherapy and BM after definitive surgery using data from the National Cancer Database from 2010 to 2016 (24). Similarly, they found that exposure to ICIs was associated with improved OS (HR: 0.62), with variable outcomes for patients with different types of tumor, although the number of cases who received immunotherapy was small (*n* = 183). Thus, we conclude that exposure to ICIs prolongs OS for patients with BM overall; however, the efficacy of this type of therapy may be cancer specific.

## Limitations of the Study

This study has several limitations, due to its retrospective design. First, our dataset has a lack of detailed tumor burden information, particularly intracranial tumor burden, which is an important prognostic factor contributing to patient survival. Second, several other important clinical information is absent. For example, percentage of symptomatic BM, extracranial tumor status, number of resection, and use of steroid could all be important prognostic factors. Third, although the database includes information on patient history of radiotherapy, it does not include the types and timing of radiotherapy. In addition, the sequence of radiotherapy and administration of ICIs is deficient. Especially, several studies have reported significant impacts on survival outcomes of different sequences of ICIs and radiotherapy (25, 26). Fourth, several newer PD-1/PD-L1 inhibitors were not included in this study, including cemiplimab, due to their relatively late approval. Finally, oncogenomic profiles were not included in this database, such as intra- and extracranial PD-1/PD-L1 expression. Although PD-L1 expression has been validated as a predictor of response in patients with NSCLC, its role in other types of cancer is still very controversial (27). Report from Goldberg et al. (9) showed the intracranial response was only observed in PD-L1-positive cohort. However, the sample was obtained from extracranial lesion that is generally not concordant with intracranial tissue (28). Furthermore, systemic and CNS response can be very discordant as well (29). Currently, it is unknown yet about the association of PD-L1 expression and predictive response rate of BM. Other valuable predictive or prognostic biomarkers are also lacking, despite tremendous efforts to identify such factors. The difficulty involved in accessing human BM samples is invariably a major barrier to many neuro-oncology studies. Retrospective study may not be able to fully address these questions. In the future, there is continuous need of prospective, biomarker driven, multidisciplinary, and innovative clinical trial design to overcome these barriers.

## Conclusions

In conclusion, large-scale data from TriNetX demonstrated a median OS of 12.3 months for patients with all types of cancer with BM, and of 7.1 months from development of BM. More specifically, median OS for patients with NSCLC, TNBC, melanoma, and RCC with BM were 8.7, 14.7, 17.8, and 15.6 months, respectively. We further investigated the efficacy of ICIs in patients with these malignancies, using cohorts matched for baseline characteristics. The results suggest that ICIs are effective for prolonging OS of patients with NSCLC, TNBC, and melanoma; however, this may not be the case in RCC, indicating that the antitumor immune effects of ICIs may be cancer specific. Further studies of underlying molecular mechanisms, better understanding of the intracranial immune microenvironment, and innovative clinical trial design are warranted to further improve BM management.

## Data Availability Statement

The raw data supporting the conclusions of this article will be made available by the authors, without undue reservation.

## Ethics Statement

This study was a retrospective study of patients from de-identified databases. It was conducted in accordance with the Declaration of Helsinki. The protocol was approved by Institutional Review Board at CAMC (IRB Number: 20-662). For this type of study, formal consent was not required.

## Author Contributions

WD, CS, and LM designed and conceptualized this study. WD, CS, and LM conducted the literature research and wrote the manuscript. All authors contributed to the article and approved the submitted version.

## Funding

The research was supported by the NIGMS of the National Institutes of Health under award number 2U54GM104942-02.

## Author Disclaimer

The content is solely the responsibility of the authors and does not necessarily represent the official views of the National Institutes of Health.

## Conflict of Interest

The authors declare that the research was conducted in the absence of any commercial or financial relationships that could be construed as a potential conflict of interest.

## Publisher’s Note

All claims expressed in this article are solely those of the authors and do not necessarily represent those of their affiliated organizations, or those of the publisher, the editors and the reviewers. Any product that may be evaluated in this article, or claim that may be made by its manufacturer, is not guaranteed or endorsed by the publisher.
